# Supplementary material for: Identifying Dopamine D3 Receptor Ligands through Virtual Screening and Exploring the Binding Modes of Hit Compounds
Source: Molecules. 2023 Jan 5;28(2):527. doi: 10.3390/molecules28020527 (PMC9862751; doi:10.3390/molecules28020527)
Supplement: Supplementary file 1 [file molecules-28-00527-s001.zip › molecules-2090693-supplementary.pdf]

Identifying dopamine D3 receptor ligands through virtual screening and exploring the binding modes of hit compounds

Hongshan Jin[a],Chengjun Wu[a], Rui Su[a], Tiemin Sun [a]*, Xingzhou Li[b]**, Chun Guo[a]*.

a Key Laboratory of Structure-Based Drug Design and Discovery, Shenyang Pharmaceutical University, Ministry of Education, Shenyang 110016, China

b Beijing Institute of Pharmacology and Toxicology, Beijing 100850, China.

Supplementary Material Document

Contents

Figure S1. The Structure, ID number, AutoDock Vina Score of the 37 compounds retained by virtual screening

Figure S2. The RMSD of D3R protein Cα atoms (blue) and Lig fit on Prot (garnet line) of MD simulations of using these top metadynamic-recorded conformations as original conformation

Figure S1. The Structure, ID number, AutoDock Vina Score of the 37 compounds retained by virtual screening.

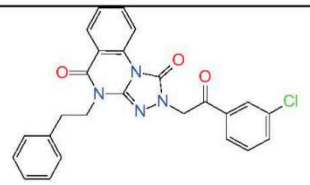<br>Autodock Vina Score: 13.4<br>IDNUMBER: F366 0225	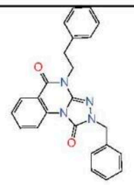<br>Autodock Vina Score: 12.6<br>IDNUMBER: F366 0245	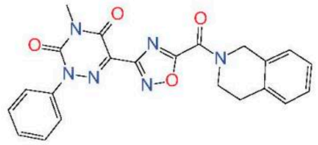<br>Autodock Vina Score: 12.0<br>IDNUMBER: F486 0373
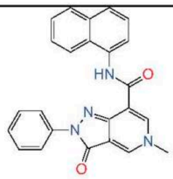<br>Autodock Vina Score: 13.0<br>IDNUMBER: F611 0038	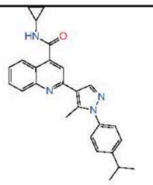<br>Autodock Vina Score: 11.8<br>IDNUMBER: F612 0729	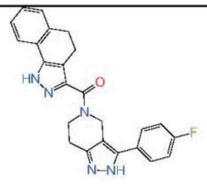<br>Autodock Vina Score: 14.0<br>IDNUMBER: F788 0161
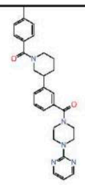<br>Autodock Vina Score: 12.1<br>IDNUMBER: F925 1017	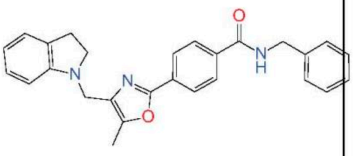<br>Autodock Vina Score: 11.8<br>IDNUMBER: G373 0280	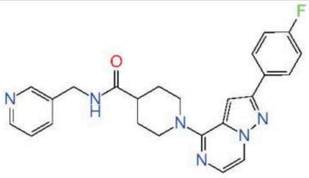<br>Autodock Vina Score: 14.0<br>IDNUMBER: G435 0137
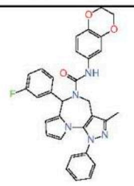<br>Autodock Vina Score: 12.9<br>IDNUMBER: G544 1316	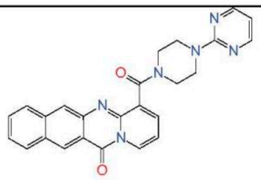<br>Autodock Vina Score: 13.6<br>IDNUMBER: K306 0682	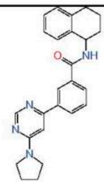<br>Autodock Vina Score: 12.4<br>IDNUMBER: L011 0060
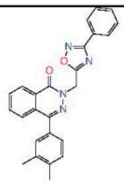<br>Autodock Vina Score: 13.9<br>IDNUMBER: L100 0151	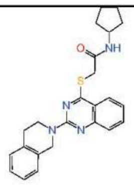<br>Autodock Vina Score: 13.3<br>IDNUMBER: L112 0768	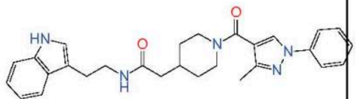<br>Autodock Vina Score: 12.3<br>IDNUMBER: L153 0098
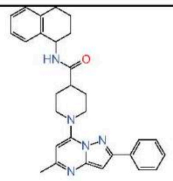<br>Autodock Vina Score: 12.5<br>IDNUMBER: L227 1012	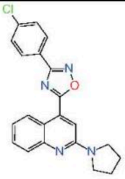<br>Autodock Vina Score: 13.8<br>IDNUMBER: L759 0276	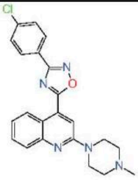<br>Autodock Vina Score: 13.2<br>IDNUMBER: L759 0287

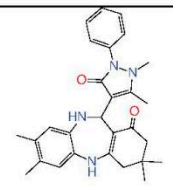

Autodock Vina Score: 14.1
IDNUMBER: 6364 0073

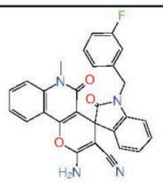

Autodock Vina Score: 13.0
IDNUMBER: 8017 6887

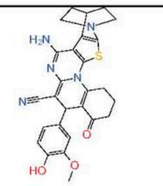

Autodock Vina Score: 12.4
IDNUMBER: 8018 0047

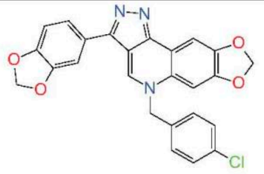

Autodock Vina Score: 11.8
IDNUMBER: C645 0112

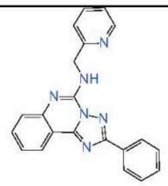

Autodock Vina Score: 12.5
IDNUMBER: C736 0093

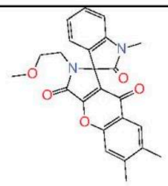

Autodock Vina Score: 13.3
IDNUMBER: D063 1105

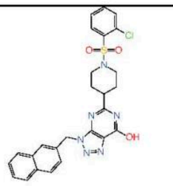

Autodock Vina Score: 13.1
IDNUMBER: D122 0034

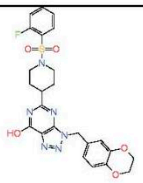

Autodock Vina Score: 12.8
IDNUMBER: D122 0078

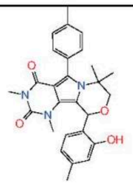

Autodock Vina Score: 12.2
IDNUMBER: D280 0447

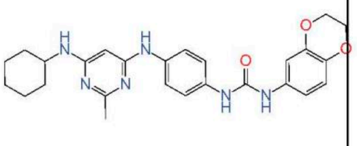

Autodock Vina Score: 12.0
IDNUMBER: D638 0102

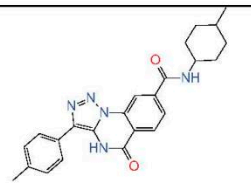

Autodock Vina Score: 12.1
IDNUMBER: E543 0286

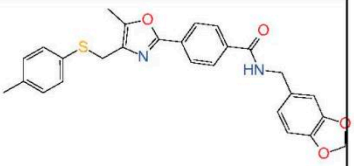

Autodock Vina Score: 11.8
IDNUMBER: E776 0059

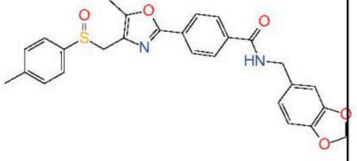

Autodock Vina Score: 12.5
IDNUMBER: E776 1501

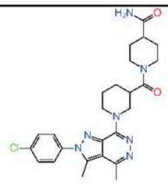

Autodock Vina Score: 13.1
IDNUMBER: E859 1320

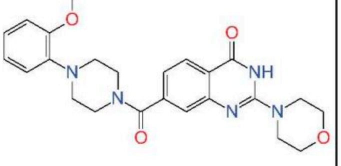

Autodock Vina Score: 11.8
IDNUMBER: F072 0025

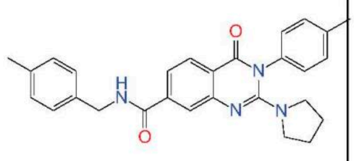

Autodock Vina Score: 12.4
IDNUMBER: F072 0905

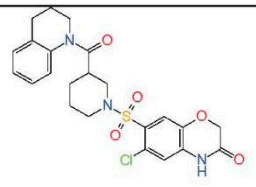

Autodock Vina Score: 12.1
IDNUMBER: F184 0480

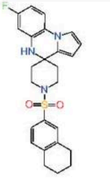

Autodock Vina Score: 13.5
IDNUMBER: F351 0364

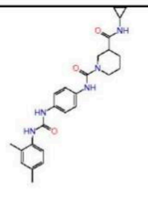

Autodock Vina Score: 11.9
IDNUMBER: M667 0989

Figure S2. The RMSD of D3R protein Cα atoms (blue) and Lig fit on Prot (garnet line) of MD simulations of using these top metadynamic-recorded conformations as original conformation

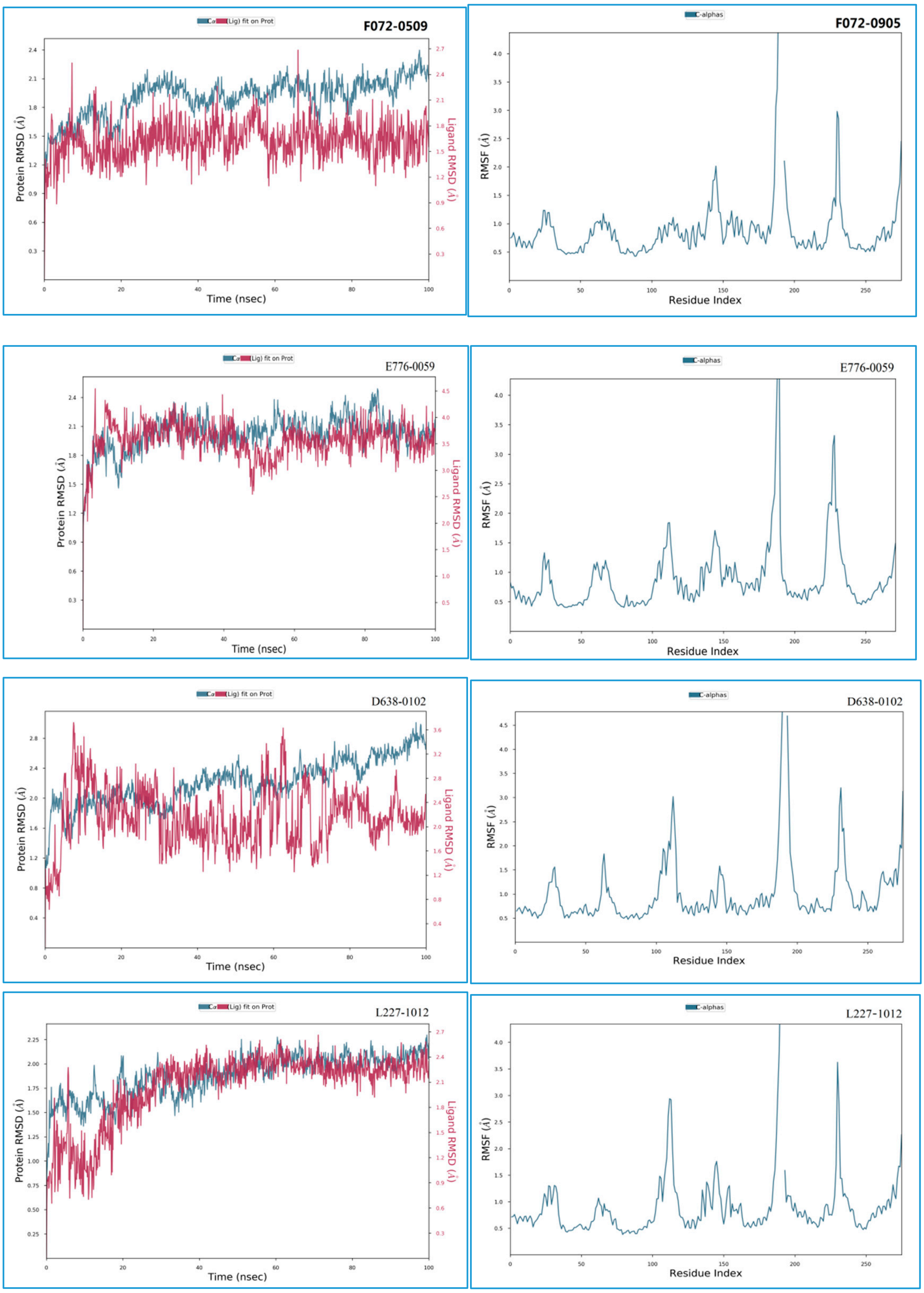

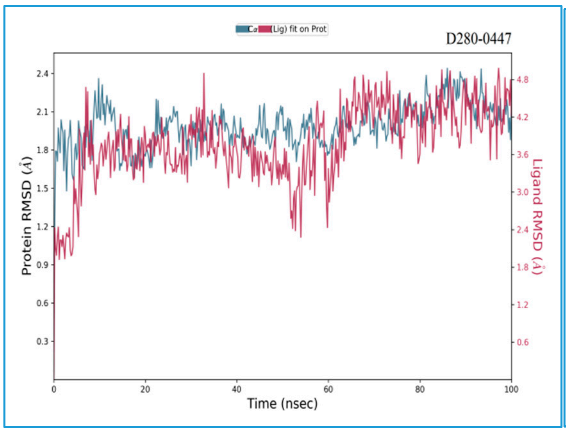

Ca (Lig) fit on Prot
D280-0447
Protein RMSD (Å)
Ligand RMSD (Å)
Time (nsec)

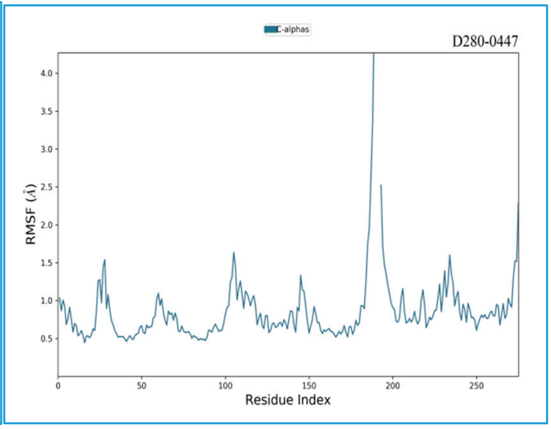

C-alphas
D280-0447
RMSF (Å)
Residue Index
